# Diversity of *Mycobacterium tuberculosis* Isolates from New Pulmonary Tuberculosis Cases in Addis Ababa, Ethiopia

**DOI:** 10.1155/2012/892079

**Published:** 2012-11-22

**Authors:** Adane Mihret, Yonas Bekele, Andre G. Loxton, Annemie M. Jordan, Lawrence Yamuah, Abraham Aseffa, Rawleigh Howe, Gerhard Walzl

**Affiliations:** ^1^Immunology Unit, Armauer Hansen Research Institute, P.O. Box 1005, Addis Ababa, Ethiopia; ^2^Department of Microbiology, Immunology and Parasitology, School of Health Sciences, Addis Ababa University, Addis Ababa, Ethiopia; ^3^Division of Molecular Biology and Human Genetics, DST/NRF Centre of Excellence for Biomedical Tuberculosis Research, MRC Centre for Molecular and Cellular Biology, Faculty of Medicine and Health Sciences, Stellenbosch University, P.O. Box 19063, Francie van Zijl Drive, Tygerberg 7505, South Africa

## Abstract

Understanding the genetic diversity of *Mycobacterium tuberculosis* is needed for a better understanding of the epidemiology of TB and could have implications for the development of new diagnostics, drugs, and vaccines. *M. tuberculosis* isolates were characterized using spoligotyping and were compared with the SpoIDB4 database of the Pasteur Institute of Guadeloupe. A total of 53 different patterns were identified among 192 isolates examined. 169 of the isolates were classified into one of the 33 shared SITs, whereas the remaining 23 corresponded to 20 orphan patterns. 54% of the isolates were ascribed to the T family, a family which has not been well defined to date. Other prominent families were CAS, Haarlem, LAM, Beijing, and Unknown comprising 26%, 13%, 2.6%, 0.5%, and 2.1%, respectively. Among HIV-positive patients, 10 patterns were observed among 25 isolates. The T (38.5%), H (26.9%), and CAS (23.1%) families were the most common among HIV-positive individuals. The diversity of the *M. tuberculosis* strains found in this study is very high, and there was no difference in the distribution of families in HIV-positive and HIV-negative TB patients except the H family. Tuberculosis transmission in Addis Ababa is due to only the modern *M. tuberculosis* families (CAS, LAM, T, Beijing, Haarlem, and U).

## 1. Introduction

Tuberculosis (TB) continues to be a major public health problem in Ethiopia. Currently Ethiopia is rated seventh among the 22 high-TB-burdened nations of the world, with a prevalence of 394 per 100,000 population in the year 2010 [1]. This situation has been worsened by the country's HIV/AIDS epidemic and emerging Multidrug-Resistant (MDR) TB. HIV prevalence was 3.5% in 2009 and among TB patients 15% were coinfected with HIV and the rate of MDR is 1.6 in new cases and 12% in retreatment cases [2]. Despite the high-TB burden in the country, very limited information is available on the genetic diversity of *M. tuberculosis* strains and the impact of HIV disease on this diversity.

Molecular typing techniques have been extensively used to speciate strains of *M. tuberculosis* involved in TB infections, studying molecular epidemiology of *M. tuberculosis*, providing insights into dissemination dynamics, evolutionary genetics, and detection of suspected outbreaks and person-to-person transmission [3]. Although recent studies are recommending the use of robust markers such as single nucleotide polymorphisms (SNP) or large sequence polymorphisms (LSP) for a better understanding of strain lineages [4], Insertion sequence (IS) 6110 restriction fragment length polymorphism (IS6110 RFLP) has in the past served as a gold standard typing method [5, 6]. However, it is costly, time consuming, and not easily standardized across laboratories.

An alternative technique is a PCR-amplification-based technique, spacer oligonucleotide typing (spoligotyping), which analyses polymorphisms of direct repeat (DR) regions. The technique is relatively simple, quick, and reliable. The method has been extensively used for simultaneous detection and typing of *M. tuberculosis* [7]. Currently spoligotyping has been successfully applied to understand the emerging problem of multi-drug-resistant (MDR) and extensively drug-resistant (XDR) TB and to investigate the virulence of certain epidemic strains of *M. tuberculosis *(such as the Beijing strain), as well as to better comprehend the epidemiology of TB and TB-HIV coinfection [8–14]. A recent study on molecular analysis of *M. tuberculosis* in Ethiopia described the diversity of strains although it failed to address the pattern in TB-HIV coinfection [15]. The present study aimed to provide additional insight into the biodiversity of clinical isolates in HIV-positive and HIV-negative patients in Addis Ababa, Ethiopia using spoligotyping and to compare the patterns obtained with those available in the international spoligotyping database, SpolDB4.0 of the Pasteur Institute of Guadeloupe.

## 2. Materials and Methods

### 2.1. *M. tuberculosis *Strains and DNA Isolation

A total of 192 *M. tuberculosis *isolates were collected between July 2009 and June 2010 from smear positive new TB cases at 4 different health centers in Addis Ababa. Demographic, epidemiologic, and clinical information for all patients was collected using a prestructured questionnaire, including sex, age, and contact (family/close contact) data. We obtained institutional and national ethical clearance. The participants agreed to participate in the study and signed a free informed consent form. The modified Petroff's method was used to digest and decontaminate the sputum specimens. An aliquot of 100 *μ*L of the sample was then inoculated onto two Löwenstein-Jensen (LJ) slants. Bacterial growth was read every week up to 8 weeks. Cultures with no growth after the eighth week were considered negative. *M. tuberculosis *isolates were identified using PCR-based genotyping with previously described methods for RD9 deletions [16]. Mycobacterial genomic DNA was extracted by heating the isolates at 80°C for 60 min and was stored at −20°C until needed for spoligotyping. 

### 2.2. Spoligotyping

Spoligotyping was carried out using the commercially available kit from Ocimum Biosolutions, India, according to the manufacturer's instructions. Briefly, the direct-repeat (DR) region was amplified with primers DRa (biotinylated at the 5′ end) and DRb, and the amplified DNA was hybridized to inter-DR spacer oligonucleotides covalently bound to a membrane. DNA from *Mycobacterium bovis *BCG and *M. tuberculosis *H37Rv were used as positive controls, whereas autoclaved ultrapure water was used as a negative control. The amplified DNA was subsequently hybridized to a set of 43 oligonucleotide probes by reverse line blotting. The presence of spacers was visualized on film as black squares after incubation with streptavidin-peroxidase and detected with the enhanced chemoluminescence system detection liquid (Amersham, Little Chalfont, United Kingdom). 

### 2.3. HIV Testing

All participants were tested for antibodies to HIV-1 and -2 after pre- and posttest counseling using rapid test (Stat pack, KHP and Unigold as a tie braker) as per the Ethiopian National guide line.

### 2.4. Database Comparison

The spoligotyping results were prepared in octal and binary formats into Microsoft Excel spreadsheets; spoligotype patterns were designated as 43-character-long strings consisting of black and white squares representing the presence or the absence of an individual spacer, respectively. The spoligo patterns which were prepared in binary and octal were entered and determined by comparing the spoligotyping results with already existing designations in the international spoligotyping database, SpolDB4.0 [17] (http://www.pasteur-guadeloupe.fr:8081/SITVITDemo/). In this database, two or more patient isolates sharing identical spoligotype patterns are defined as SIT (spoligotype international type) whilst single spoligo patterns are defined as “orphan” isolates. The SpolDB4 defines 62 genetic families/subfamilies and includes specific signatures for various *M. tuberculosis* complex members such as *M. bovis, M. caprae, M. microti, M. canettii, M. pinipedii, and M. africanum*, as well as including rules for defining the major families/subfamilies for *M*. *tuberculosis*. At the time of matching analysis, the updated SpolDB4.0 contained 39,609 patterns distributed among 2,881 shared types in 121 countries. Patterns that were not found in SpolDB4.0 were assigned to families and subfamilies using the SpotClust program, which was built on the SpolDB3 database (http://cgi2.cs.rpi.edu/_bennek/SPOTCLUST.html). 

## 3. Result

### 3.1. Demographic Information

Of the total 192 patients sampled, 26 (13.5%) were HIV positive, 136 (70.8%) were HIV negative and the remaining 30 (15.6%) were not tested for HIV. The mean age was 28.7 years (range 18–72). There were slightly more male participants than female (56.9%). For the HIV-positive patients, the mean CD4-cell count at the time of presentation was 210 ± 23.9 cells/*μ*L. 

### 3.2. Genetic Diversity and Family Assignment

**Figure 1:**
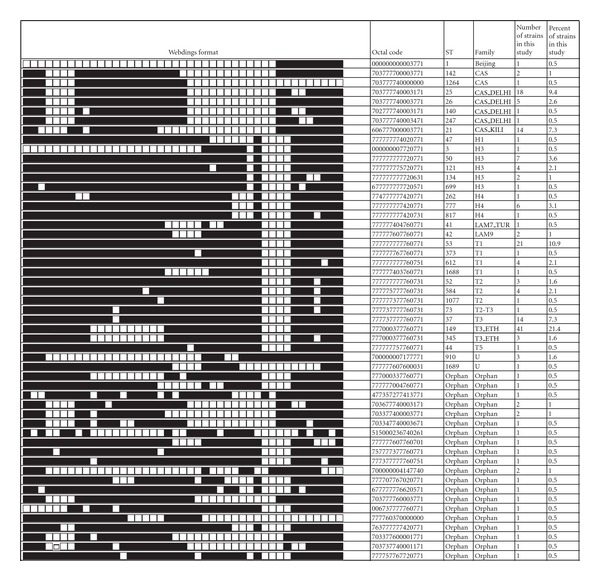
Spoligotype pattern of clustered *M. tuberculosis* strains. The black and white boxes indicate the presence and absence, respectively, of the specific spacer at position 1 to 43 in the DR locus. CAS = Central Asian; LAM = Latin American-Mediterranean; H = Haaralam, and U = Unknown.

Among the 192 typed isolates, 169 (88%) were classified into one of the 33 shared international types (SITs) according to SpolDB4.0. The remaining 23 isolates generated 20 different spoligotypes with 3 new shared spoligotypes (*n* = 2 in each cluster) that had not been previously described in the database. Among the total 53 spoligotype patterns characterized in the present study, 20 patterns (including the 3 new clustered patterns) corresponding to clusters with 2–41 isolates per cluster were identified, accounting for a very high clustering rate of 88% (169/192). Out of the 33 patterns that did not form clusters, 17 represented true orphan patterns that did not previously exist in SpolDB4.0 and 16 represented pseudoorphans which were present as singles in this study but found in SpolDB4.0 (the ST and Family distribution of clustered strains is summarized in Figure 1).

Family assignment revealed that the T family constitutes the most predominant family in our study, constituting of 104, (54.2%) isolates with the following distribution: T3_ETH (44, 22.9%); T1 (34, 17.7%), T2 (8, 4.2%); T2-T3 (1, 0.5%), T3 (16, 8.3%), and T5 (1, 0.5%). The CAS families (48, 26%) were the second dominant families with CAS1_DELHI (25, 13%); CAS_KILLI (14, 7.3%) and CAS (9, 4.7%). Other families found were Haarlem (H) (24, 13.2%) with the following distribution: H1 (1, 0.5%), H3 (15, 7.8%), H4 (recent results suggest that the H4 sublineage belongs to a new “Ural” lineage that is distinct from Haarlem) (8, 4.2%); the LAM Family (5, 2.6%): LAM9 (4, 2.1%), LAM7_TUR (1, 0.5%); the Beijing family (1, 0.5%); family 34 (1, 0.5%); family 36 (1, 0.5%) and 4 (2.1%) strains were undetermined (the main results of the spoligotyping analysis are summarized in Table 1). 

**Figure 2:**
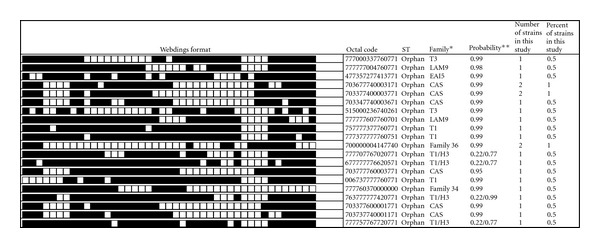
Spoligotype pattern of orphan *M. tuberculosis* strains from HIV-positive and HIV-negative patients. *SpotClust-program-assigned family. **Probability that the spoligotype pattern belongs to the family.

Among the total 32 clustered isolates, a total of 126 isolates (65.6%) from 8 different phylogenetic clusters formed the major isolates and 43 isolates (22.4%) from 25 clusters (containing sixteen shared types contained only one patient, three shared types contained only two patients each, three shared types contained only three patients, and two shared type contained four patients) formed the minor isolates. Sixteen isolates were pseudoorphans where as 23 isolates generated 20 unique spoligotypes that had not been previously described in the database. The spoligotype pattern of the orphan isolates is summarized in Figure 2.

**Figure 3:**
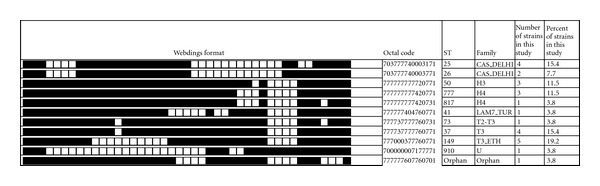
Spoligotype pattern of *M. tuberculosis* strains from HIV-positive subjects.

For the HIV-positive patients, spoligotyping produced a total of 11 different patterns. Among the 26 patients, 25 (92.3%) were classified into one of the 10 shared international types (SITs) according to SpolDB4.0. The remaining 1 isolate generated a unique spoligotype pattern that had not been previously described in the database. Among the total 11 spoligotype patterns characterized in the present study, 5 patterns corresponding to clusters with 2–5 isolates per cluster were identified and the remaining 5 patterns were pseudoorphans. One spoligo pattern represented true orphan pattern that did not previously exist in SpolDB4.0. Family assignment in HIV-positive subjects also revealed that the ill-defined T family constitutes the most predominant family (38.5%) followed by H. Family constituting 26.9% and CAS_DELHI (23.1%). Although the small sample size of HIV-positive subjects did not allow us to make a strong comparison with HIV-negative subjects, we did not find a significant difference in the distribution of the different families in the two groups. The spoligo pattern of the isolates from HIV-positive subjects is summarized in Figure 3.

## 4. Discussion

Analysis and timely updates of the *M. tuberculosis* strain distributions in a given country and comparisons to worldwide patterns provides insights into transmission mechanisms, emergence of drug resistance and particularly virulent strains, and information relevant to the development of new diagnostics, drugs, and vaccines.

This study has been undertaken in HIV-positive and HIV-negative patients in order to gain a better understanding into the population structure of *M. tuberculosis *in Addis Ababa. Spoligotyping was used as a primary typing tool because of its ease of use, straightforward coding and international database of global isolates for comparative analysis. 

**Figure 4:**
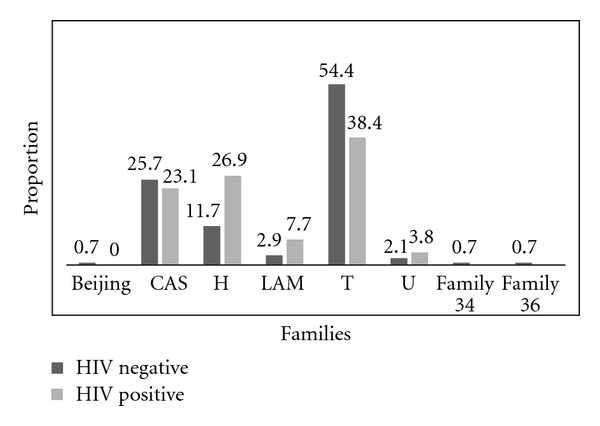
*M. tuberculosis* spoligotype families in HIV-positive and HIV-negative patients.

Distribution of the predominant clades of *M. tuberculosis* strains shows variation among different populations [17]. In our study, the majority of the isolates (83.2%) belonged to three major families: T family (54.2%), CAS (26%) and H (13.2%) and there is no difference in the distribution of families in HIV-positive and HIV-negative subjects (Figure 4). 

Although the T family is the most prevalent in this study, it is believed that this family does not represent a clade in a strict evolutionary sense, because it includes by default strains that could not be classified in one of the established families with well-established phylogeographical specificity [17]. Hence it is frequently referred to as the “ill-defined” family of *M. tuberculosis *that is found worldwide. 

The T3_ETH is the dominant family, and previous reports also showed this spoligotype present in high proportion in Ethiopia [15] and it is believed to be specific for Ethiopia and this ST is rarely reported in other countries [17]. The CAS family, which is the second most frequent spoligotype in this study, had been reported in previous studies in Ethiopia [15] and also prevalent in Tanzania [18] with CAS1_KILI the predominant and in Kenya 35.6% of 73 isolates were of the CAS family [19]. The H family constituted 13.2% of the total family in our study and its wide spread in different geographical regions of the world such as Asia, Europe, Middle East, and Africa has been documented with some reports associated with outbreaks in Argentina [20] and in Czech Republic [21]. 

The other families were LAM and Beijing comprising 5 and 1 isolates, respectively. Among the LAM family we have two strains, LAM7_TUR and LAM9. The LAM7_TUR is believed to be restricted strain in Turkey [22] where as LAM9 has been reported from different parts of the world including Africa. SIT1 corresponding to the Beijing genotype is the first report from Ethiopia but it needs to be confirmed with other molecular methods as spoligotyping has limitations for genotypic classification. The Beijing family has spread globally during recent years, and is seen as an indicator strain for recent import of *M. tuberculosis *into a setting. In recent years several countries reported an increase over time of the proportion of TB due to Beijing genotype strains including countries in the region [18, 19]. 

## 5. Conclusion

Despite the presence of predominant shared types, the diversity of the *M. tuberculosis* found in the present study is very high; 192 isolates produced 53 different spoligotypes. The families observed in this study were more than 85.5% of the total 62 families currently registered in the fourth international spoligotyping database, SpoIDB4. Spoligotyping has less discriminatory power; therefore, for a better understanding and confirmation of the diversity, more discriminative techniques will be required. Moreover, the effect of this diversity on *M. tuberculosis* transmission and whether strain differences which might elicit different immune responses is not clear and it needs further study. Different studies have indicated *M. tuberculosis* strains differ in their immunogenicity and virulence [23–25]. It is also advisable to do followup studies combined with contact-tracing and epidemiological linking in order to obtain a clear molecular-epidemiological overview of the Beijing and other strains. This study was done in a few health centers in the capital city of Addis Ababa, so our findings here may not be representative of the entire country; therefore, we recommend future wider studies with a better molecular method with a better discrimination power like single nucleotide polymorphism (SNP) typing and mycobacterial interspersed repetitive units—variable number of tandem repeats (MIRU-VNTR) to elucidate strain diversity in detail within Ethiopia. 

## Figures and Tables

**Table 1:** Family and family distribution and associated ST for each isolate in the study.

Family	Family	Shared type, number of isolates
Beijing *n* = 1 (0.5%)	Beijing *n* = 1	1, *n* = 1

CAS *n* = 50 (26%)		142, *n* = 2
	CAS *n* = 9	1264, *n* = 1
		Orphan, *n* = 8
	CAS1_KILI *n* = 14	21, *n* = 14
		25, *n* = 18
	CAS_DELHI *n* = 25	26, *n* = 5
		140, *n* = 1
		247, *n* = 1

H *n* = 24 (13.2%)	H1 *n* = 1	47, *n* = 1
		3, *n* = 1
		50, *n* = 7
	H3 *n* = 15	121, *n* = 4
		134, *n* = 2
		699, *n* = 1
		262, *n* = 6
	H4 *n* = 8	777, *n* = 1
		817, *n* = 1

LAM *n* = 5 (2.6%)	LAM7_TUR *n* = 1	41, *n* = 1
	LAM9 *n* = 4	42, *n* = 2
		Orphan, *n* = 2

T *n* = 104 (54.2%)		53, *n* = 21
		373, *n* = 1
	T1 *n* = 34	612, *n* = 4
		1688, *n* = 1
		Orphan, *n* = 7
		52, *n* = 3
	T2 *n* = 8	584, *n* = 4
		1077, *n* = 1
	T2-T3 *n* = 1	73, *n* = 1
	T3 *n* = 16	37, *n* = 14
		Orphan, *n* = 2
	T3_ETH *n* = 44	149, *n* = 41
		345, *n* = 3
	T5 *n* = 1	44, *n* = 1

EAI *n* = 1 (.5%)	EAI5	Orphan, *n* = 1

Family 34 *n* = 1 (0.5%)		Orphan, *n* = 1

Family 36 *n* = 2 (1%)		Orphan, *n* = 2

U *n* = 4 (2.1%)	U *n* = 4	910, *n* = 3
		1689, *n* = 1
